# The Foreign Journals: Anatomy and Physiology

**Published:** 1842-04

**Authors:** 


PART THIRD.

### Selections from the British and Foreign Journals.

### I. THE FOREIGN JOURNALS.

### ANATOMY AND PHYSIOLOGY.

*On the Formation of Contagious Confervæ on Living Frogs.*
By Dr. Stilling, of Cassel.

The object of this paper is to prove that the formation of conferva-like tubes on frogs is not to be regarded, as it is by Hannover, as the result of a vegetable contagion growing in the water, but is nothing more than the development of infusoria from ova, which, being most probably derived from external sources, come in contact with an organic substance that is undergoing decomposition, and then become the seats of new vital processes.

The author says he saw the conferva-like tubes just as Hannover [Brit. and For. Med. Rev., vol. IX. p. 550] describes them. The black granules were at first contained within the tubes, but afterwards were extracted from them and adhered in tufts to their exterior. By long and careful observation he saw how, in the tubes which inclosed the granules, all was at first unmoved, but soon, at various intervals within them, rapid currents began to move in them, as if certain parts of a firm gelatinous substance had suddenly become very thinly fluid; and how this fluid made for itself a way through one end of the tube, and flowed out of it. The black granules were moved in various quantity with these currents, and often flowed with them out of the tubes; but the most striking phenomenon was the independent motion of the black granules, of the reality of which, as well as of its difference from the ordinary Brownian-molecular motion, the author and many others repeatedly convinced themselves.

These granules were repeatedly seen to move pretty rapidly in all directions, through certain spaces of the tube, but not commonly through its whole length at once. It appeared as if the contents of the tubes at the parts which contained the moving granules were more fluid than at the parts where the granules lay immoveable, as if imbedded in a denser jelly. A heap of these granules often vibrated very vividly, but still the motions of every individual granule could be observed separately from those of the rest, even though its movements were not considerable. Often too the granules were seen like little infusory animalcules moving from one wall of the conferva-like tube to the other, upwards or downwards, in short, in all possible directions through an extent of between one and two lines; and, although no locomotive organs could be seen, yet their motions were quite regular, and just such as would be produced by a regular motor apparatus, like that of mites. Nor were they confined to the interior of the tubes; they were seen to be still actively continued in portions of the jelly-like fluid that had been forced out of the tubes.

Further examination proved that in every conferva-like tube whose contents were in part evacuated, there was an innumerable multitude of small, straight, hair-like, semi-transparent, linear animalcules, apparently about a line and a half long, which presented an uncommonly vivid vibratory motion, and which usually lay with one end (the head) against the wall of the tube, and stretched out their bodies and tails at right angles away from it. They seemed, indeed, as if they had bored with their heads into the substance of the tube and derived their nourishment from it, while they were making the rapid vibratory motions with their bodies. They commonly adhered in heaps to one or more spots, or to the greater part of the circumference of a tube, and in the latter case they made it look as if it were beset with the finest, semi-transparent, whitish, needle-like crystals, or as if it were lined with portions of the finest glass-thread a line or two long. Their manifold motions clearly showed that they were living infusory animalcules.

On almost every occasion it was observed that some of the animalcules were swimming about, each in connexion with one of the black granules that are contained in the conferva-like tubes. And this was so often seen that the author does not hesitate to regard the granules as the ova from which the hair-like infusoria are produced; nor to consider that the movements of the former depend on the latter being hatched and forcing their way from their cells, the last stage of the hatching being that in which the animalcule is seen, as just mentioned, moving about with one of the black granules appended to his tail.

But the degree of development hitherto traced out does not yet afford a complete history of these granules and animalcules. If the efflorescence from the frogs' toes, which presents the phenomena just described between the second and fourth days, be examined at a later period, many of the animalcules are found to be grown to three or four times their original size, but they are now bent into a horse-shoe shape, and at both their extremities have dark spots like the original black granules, but depending on the doubling up of their very ends. But at this time also there are yet a greater number of serpent-like infusoria of different sizes moving about, and the number of the original smaller vibrating animalcules is much diminished, or they have altogether disappeared. The author does not hesitate to regard the larger animalcules, which have the shape and rapid tortuous movements of snakes, as the representatives of the more perfect stage of development of the smaller hair-like kind. As soon as they make their appearance nothing but traces can be discovered of the conferva-like tubes; they seem by this time to have been completely broken up.

The author made several experiments on inoculation with these creatures. Small wounds were made on the backs of several salamanders, and into each was put a small quantity of the efflorescence from the frogs' toes, (i. e. from the toes of frogs on which the conferva-like tubes and their animalcular contents had been spontaneously produced after destruction of the lower part of the spinal cord). On the second day the wounds were covered with a whitish substance, which gradually spread; several of the salamanders died and all became sickly. This white substance consisted of conferva-like tubes just like those that had been inserted in the wound; after four or five days it was all thrown off from the wound, complete cicatrization took place, and the animal recovered its health. Dead flies, placed in the same vessels with frogs that had any of this efflorescence on them, soon became covered with it without the aid of inoculation. A toad, into whose wounded back a toe thus affected and cut off a frog's foot was introduced, was found dead next day. On the whole the inoculation was more successful in salamanders than in frogs; among the latter it never succeeded unless they were previously sickly; the matter from dead animals was as efficient as that from the living.

To determine whether the formation of the conferva-like tubes were the result of putrefaction, or whether it were connected with the putrefaction of the blood, or produced from the latter or from the nervous substance, or derived entirely from other sources, the author instituted some further experiments. Dead frogs which had not during life been affected with the peculiar disease, were left to putrefy in water. In many, the conferva-like formation was produced and went through all its stages; in others, no trace of it could be found; but in all, as soon as the putrefaction had begun, a white granular substance formed in a layer on the surface of the water, which was found to contain the same black granules as were seen in the conferva-like tubes, the same serpent-like animalcules as had been found in the older conferva-like tubes, and the same islands of gelatinous animal matter, with the black granules and vibrating infusoria as had been already constantly seen and described. The same productions were found in films on the water in which frog's blood and nervous substance had been laid, although in neither case was there any production of the conferva-like tubes.

In his summing up the author concludes that the material which has been described as composed of confervæ is really not a vegetable, but an animal exudation taking place from the blood-vessels of a diseased part, in which, from defective nervous influence, or some other cause, the blood has stagnated, or moves very slowly. He cannot account for its taking the form of tubes, nor for some of the other anomalies which it shows in the kind of animals and circumstances in which it chiefly occurs; but, once formed, it constitutes the most favorable nidus for the development of the ova of this particular infusory animalcule; which ova, like those of other infusoria, must be regarded as constantly present among us in great numbers, wanting only an appropriate nidus in which to commence their further growth.

*Muller's Archiv.* Heft iv. 1841.

---

*On the Development of the Hair.* By Dr. GUSTAV SIMON, of Berlin.

THE author's investigations were for the most part made on fœtal pigs. In those that were not more than two inches long, he found, as Heusinger did, that the skin had the appearance of having been sprinkled with fine black dust, or if not so, that it contained white cells of the same form as those from which, in young black or spotted pigs, the dark spots proceeded. These cells, whether black or white, were found to be the embryonic hair-follicles; they lay very obliquely or almost horizontally beneath the surface of the skin, and were somewhat pear-shaped, their smaller ends being next the surface. They were lined by small granules, probably the nuclei of cells, and (in the parts that would have black hairs) by a layer of black pigment in round or star-shaped cells. It was obvious that the hair-follicles are formed in the embryo earlier than the hairs, for in fœtuses so small as these no trace of a hair could be seen.

The first part of a hair that could be discerned was the root, which appeared as a small mass of round, and sometimes black, granules, like the cells of the rete Malpighii, set in the bottom of the follicle. From these cells are formed the shaft of the hair, probably, as Henle supposes, by the elongation and occasional swelling of their nuclei to form the fibres of the cortical substance, and by the gradual elevation of the cells of one set, by others produced after them, to form the cellular medullary substance.

When the hairs have grown to a length greater than that of the containing follicles, they do not at once grow straight out of them, but their ends bend back again so as to form loops, with their arches directed to the surface, and the very point of the hair turned back to the root. Their extrusion is prevented for a time by a thin membrane composed of very fine cells, like those of epithelium, but which is separate from the true epidermis, and must rather be regarded as the fœtal layer of the amnios. As this membrane separates, the hairs make their way out of their follicles, and by the time the fœtus is between eight and twelve inches long, they commonly all project from the skin.

The sebaceous glands attendant upon the hair-follicles are always formed rather later than they, but traces of them are visible before the root of the hair is produced.

Exactly confirmatory observations were made on several fœtal dogs and calves.

*Müller's Archiv.* Heft iv. 1841.

*Description of a hitherto-undescribed Entozoon removed from the Eye of a Horse, through an opening in the Cornea.* By Dr. A. Numan, of Utrecht.

Von Nordmann, who wrote in 1832, was acquainted with about seventy species of entozoa, of the families of nematoidea, cystica, and trematoda, which occurred in different parts of the eyes of man and other vertebrata; and since that time several other species have been discovered and described. Many examples of entozoa in the eye of the horse have been published in the various scientific and veterinary journals, and, although it is difficult to determine exactly what species is described in each case, it may be assumed that several species have already been found.

The subject of the present case was a three-year-old mare, which was found by the friend of the author suffering from severe inflammation of one of the eyes, with such an opacity of the cornea that it was with some uncertainty that he thought he could perceive a foreign body floating in the anterior chamber. After reducing the inflammation however, the presence of its cause, some entozoon moving in the eye, and especially active during bright sun-light, was no longer doubtful. This he therefore removed through a section of the lower part of the cornea, similar to that made in the ordinary operation for the extraction of a cataract. No ill consequences ensued from the operation, and in a month after it the mare was well, and had recovered her sight.

The entozoon thus removed was half an inch long, and on an average a line broad. Its body was rounded but flattened, and at tolerably regular distances seemed constricted, as if it consisted of a number of sections or joints like those of the tape-worms or the larvæ of some insects, though with the latter it could not possibly be confounded. The joints were nine in number, the anterior being somewhat longer and broader than the posterior, and the last of all blunt and somewhat clavate. On the third joint from the head, and on its abdominal surface, was a channel-like opening which might, without doubt, be regarded as the ovary connected with ducts, like those of many entozoa, and among them, of the strongylus. From this aperture a fluid was extracted, which, when examined with Schröder van der Kolk's microscope, was found to contain a great number of ova, which measured about $\frac{1}{15000}$th of an inch in length, and from $\frac{1}{20000}$ to $\frac{1}{25000}$th of an inch in thickness. The head was also blunt, and cup-shaped, with a depression in its middle where there were a number of very small, dark, horn-like points, in the midst of which was the oral aperture.

Finding no description of a worm like it in any helminthological work, and believing that it may best be referred to the genus *monostoma*, the author has proposed to call it *monostoma settenii;* its specific distinction being taken from the name of the veterinary surgeon under whose care the case occurred, and by whom the parasite was sent to him.

*Tijdschrift voor naturlijke Geschiedenis en Physiologie,* 1840. 7de Deel. 4 Stuk.

---

*On a new Account of the Movements of the Bones of the internal Ear, and of the Membrana Tympani.* By M. Bonafont.

The author's observations were chiefly made by exposing the muscles, and drawing them gently in the direction of their fibres. By acting thus on the tensor tympani, or internal muscle of the malleus, he saw the handle of that bone drawn inwards and a little forwards, stretching the posterior, and relaxing the anterior, fibres of the tympanum. By similar means he found the direct office of the stapedius muscle to be the drawing of the head of the stapes outwards and a little forwards. By this movement the incus is made to rotate around an imaginary axis, passing horizontally through the angle formed by its branches; and its anterior branches being carried backwards and a little outwards, and its upper branch upwards and a little outwards, the malleus is in its turn moved, so that its head is carried forwards and its handle backwards. By this motion the tympanum is affected in a manner exactly contrary to that which results from the contraction of the tensor tympani, for its anterior fibres are now stretched and its posterior relaxed.

The author denies the muscularity of the laxator tympani, and holds that the whole of the membrane can be made tense only when the two muscles, the tensor tympani and stapedius, act coincidently.

He has invented an instrument for examining the membrana tympani, which he calls a *speculum otique phosactéon,* and he mentions some of the facts which by its means he has observed. Among them he relates this case: "An artilleryman working at the arsenal had heated a large plate of iron, and his comrade, in taking it red-hot from the fire, carried it close by his right ear. He felt at the instant a strong sensation of heat, and a slight crack in the ear, and from that time he has had dullness of hearing, and when he speaks it seems to him as if his voice escaped through his ear, so that he instinctively puts his hand to the side of his head to prevent it. I examined him with my speculum, and distinctly perceived a slight rent at the anterior part of the membrana tympani, with a chronic inflammation of the mucous lining of the external meatus."

*Gazette Médicale. Janvier* 28, 1842.

---

### *Case of complete Anchylosis of the Temporo-maxillary Articulations.* By M. PAYAN.

IN examining the body of a man, seventy-five years old, who died in 1835 of Asiatic cholera, M. P. found so complete a union of the temporal and lower jaw-bones, that they seemed to form but one; and the osseous substance which had formed around their articulations, so completely covered them, that the line of separation between them could not be detected. All that could be learned of the patient's history was, that, at five years old, he had a violent blow on the head, after which his jaw became fixed; and that it had continued so ever since, his food having been always taken through a small aperture made by contracting his upper incisor teeth.

*Revue Médicale. Novembre,* 1841.

---

### *Remarkable Monstrosity.* By Dr. FRIESE.

THIS child, which lived only half an hour after birth, was eighteen inches long, and weighed six pounds. The external genital organs were wanting: their place was occupied by a fold of skin. The testicles were absent; but behind the abdominal rings were two small membranous vesicles, containing a limpid fluid. There was neither epididymis, vas deferens, nor vesicula seminalis; the urinary bladder was normal; the urethra terminated in cellular tissue below the symphysis pubis. In the right arm, the radius and ulna gradually separated below the elbow-joint, so that at the carpus they were nearly four inches apart. They were covered and connected by a thin, tense, transparent yellowish-white membrane, without any trace of muscles. The os pisiforme was the only bone of the carpus that was present; the hand also was absent; and the whole fore-arm was not unlike a bat's wing.

*Casper's Wochenschrift. December* 25, 1841.

---

### *Account of a Phrenological Visit to the Penitentiary for young Criminals at Paris, made by* M. VOISIN, *in Company with a Committee of Members of the Royal Academy of Medicine, on February* 17, 1839.

IN addition to M. Voisin and the committee from the Academy of Medicine, there were present MM. Boullon and Pontignac de Villars, of whom the former was governor of the prison, the latter, secretary. Four hundred young criminals were examined, one by one, by M. Voisin; who, having looked at the form of each one's head, and examined it with his hand, directed him to go to the *right* or the *left,* according as his character or natural endowments appeared to be good or bad. These he subsequently divided into four classes, putting the worst in the first, the best in the fourth, and arranging in the two intermediate series those who formed a sort of *juste milieu* between the others.

Of the 400 boys originally examined, 254 were selected by M. Voisin as those whose good or evil qualities were most distinctly marked. The fourth, or best class, contained only 25, or one tenth of the whole; while 61 were arranged in the first or worst class. Of the remaining 168, 77 were placed in the third class, 91 in the second, the bad again preponderating.

M. Boullon, the governor, then gave his evidence as to the character of the youths thus classified by M. Voisin. He stated that M. Voisin's first class included, in a very great proportion, the bad characters in the house, or those whose intellectual faculties were most limited. The second and third divisions appeared to M. Boullon not to offer any striking differences between each other; but the fourth class comprehended almost all those children who were most docile, most intelligent, and most industrious. This class included the greater number of those who were employed as monitors in the school, or as overlookers in the workshops. The testimony of M. de Villars corresponded almost completely with that of M. Boullon.

A long discussion followed the reading of the report in the academy. The two chief objections raised by the debaters were, that the testimony of the governor and secretary of the gaol was given after M. Voisin had pronounced on the characters of the boys, instead of before he had expressed his opinion; and secondly, that M. Voisin's classification implies that the intellectual and moral faculties are intimately connected, and become developed in the same proportion, while in reality no such absolute relation between mental and moral endowments exists.

*Bulletin de l'Académie Royale, Novembre,* 1841.

